# Predicting the phenotypic effects of non-synonymous single nucleotide polymorphisms based on support vector machines

**DOI:** 10.1186/1471-2105-8-450

**Published:** 2007-11-16

**Authors:** Jian Tian, Ningfeng Wu, Xuexia Guo, Jun Guo, Juhua Zhang, Yunliu Fan

**Affiliations:** 1 Biotechnology Research Institute, Chinese Academy of Agricultural Sciences, Beijing 100081, China; 2 Agricultural By-Products Processing Research Institute, Academy of Planning and Designing of the Ministry of Agriculture, Beijing 100026, China; 3 Department of Biomedical Engineering, Beijing Institute of Technology, Beijing 100081, China

## Abstract

**Background:**

Human genetic variations primarily result from single nucleotide polymorphisms (SNPs) that occur approximately every 1000 bases in the overall human population. The non-synonymous SNPs (nsSNPs) that lead to amino acid changes in the protein product may account for nearly half of the known genetic variations linked to inherited human diseases. One of the key problems of medical genetics today is to identify nsSNPs that underlie disease-related phenotypes in humans. As such, the development of computational tools that can identify such nsSNPs would enhance our understanding of genetic diseases and help predict the disease.

**Results:**

We propose a method, named Parepro (Predicting the amino acid replacement probability), to identify nsSNPs having either deleterious or neutral effects on the resulting protein function. Two independent datasets, HumVar and NewHumVar, taken from the PhD-SNP server, were applied to train the model and test the robustness of Parepro. Using a 20-fold cross validation test on the HumVar dataset, Parepro achieved a Matthews correlation coefficient (MCC) of 50% and an overall accuracy (Q2) of 76%, both of which were higher than those predicted by the methods, such as PolyPhen, SIFT, and HydridMeth. Further analysis on an additional dataset (NewHumVar) using Parepro yielded similar results.

**Conclusion:**

The performance of Parepro indicates that it is a powerful tool for predicting the effect of nsSNPs on protein function and would be useful for large-scale analysis of genomic nsSNP data.

## Background

Almost 90% of human genetic variations result from single nucleotide polymorphisms (SNPs) [1]. Among SNPs resulting in amino acid changes, non-synonymous SNPs (nsSNPs) are an important source of individual variation and can result in inherited diseases and drug sensitivity [2-4]. Therefore, the identification of nsSNPs that affect protein function and relate to disease will be a challenge in the coming years [3,5-8].

A variety of methods have been developed to identify whether an nsSNP is detrimental to protein function *in vitro*. Most of these methods utilize evolutionary data [3,8-17], protein structure information [2,18,19], or both [2,7,20-22]. Ng and Henikoff [8,16,23] developed the software SIFT (Sorting Intolerant from Tolerant) to predict the effect of nsSNPs on protein function; SIFT is based on sequence conservation and scores from position-specific scoring matrices. Some studies [24-26] have used phylogenetics to identify functionally critical residues within a protein. The MAPP (Multivariate Analysis of Protein Polymorphism) [18] software exploits the physicochemical variation between wild-type amino acid residues and newly introduced residues to identify nsSNPs that impair protein function. The method Align-GVGD [9] uses both genetic biochemical variation and genetic distance between the wild-type residue and newly introduced residue to predict the effects of an nsSNP. Some methods [2,20-22] take advantage of three-dimensional structural information to analyze the impact of amino acid changes on protein function. Wang and Moult [4] found that the vast majority of nsSNPs that are related to diseases affect protein stability rather than function. Specific factors that determine stability of a protein were then used to predict the effects of nsSNPs. Chen *et al*. [27] used solvent accessibility of residues to predict deleterious mutations.

Support vector machine (SVM) has gained popularity over other machine learning methods for interpreting biological data [28-35] because of their ability to very effectively handle noise and large datasets/input spaces [36,37]. Then, some methods [2,7,10,21] have been designed based on the SVM [38] to predict the effect of nsSNPs. Capriotti *et al*. [10] developed a method that depends only on the evolutionary information around the nsSNP. Peng Yue and John Moult [2] also proposed a method that uses the conservation and type of residues observed at a base change position within a protein family. Karchin *et al*. [7] and Bao *et al*. [21] introduced two methods based on structural and evolutionary information. The structural information mainly concerns areas in the protein that are buried, as well as the fraction polar secondary structure, solvent accessibility, z-score and buried charge. The evolutionary information mainly uses Hidden Markov model PHC score, Hidden Markov model relative entropy, SIFT score and the biochemical difference between the wild-type residue and newly introduced residue.

Here, we propose a method that predicts nsSNPs based on the SVM [38]. This method, named Parepro (Predicting the amino acid replacement probability) uses evolutionary information surrounding an nsSNP. In addition, properties from the AAindex [39,40] and from evolutionary information are combined to determine the dissimilarity between the wild-type and newly introduced residues. Parepro predicted the total number of nsSNPs with higher accuracy than other methods and was not dependent on structural information. In this study, two independent datasets, HumVar and NewHumVar, taken from the PhD-SNP server [10], were applied to train the model and test the robustness of Parepro, respectively.

## Results

### The nsSNP prediction performance of Parepro

**Figure 1:**
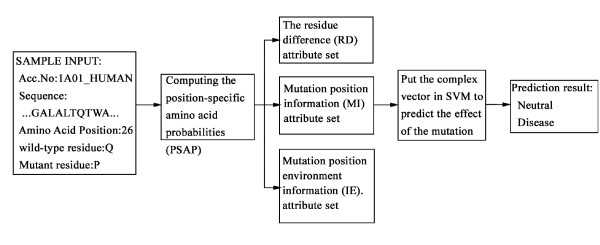
**Brief flow chart illustrating the prediction procedure of Parepro**. First, the position-specific amino acid probabilities (PSAP) of the target sequence are calculated. Second, three attribute sets are constructed using the PSAP information in combination with the RD, MI, and IE properties of the amino acids. Finally, the complex vector of Parepro is integrated and used to predict the effect of an nsSNP.

Figure 1 presents a flowchart illustrating the procedure of Parepro. Homologous sequences of a protein containing the target nsSNPs were selected from the Swiss-Prot database, aligned, and weighted. Position-specific amino acid probabilities (PSAP) of the amino acids surrounding mutation position were then estimated. Next, three attribute sets, namely residue differences (RD), mutation position information (MI), and information on the environment around the mutation position (IE) were constructed and combined. In this study, the attribute set IE was calculated from the six residues on either side of the mutation, because this was the smallest number of residues that produced accurate results. To evaluate the performance of different attribute sets, a 20-fold cross-validation test on the HumVar dataset was carried out. All variants in the HumVar dataset could be predicted by using different attribute sets.

Table 1 shows the performance of the three attribute sets when applied individually or in various combinations. The prediction performance of attribute set IE was the poorest among the three. By comparison, the performance of the other attribute sets (RD or MI) was high. Nevertheless, association of the attribute set RD or MI with IE improved performance such that the overall accuracy (Q2) and Matthews correlation coefficient (MCC) were approximately 75%, respectively. The highest prediction accuracy was obtained, however, after these three attribute sets were combined into a new vector, Parepro, suggesting that the three attribute sets reinforce each other in the analysis.

**Table 1:** The prediction performance of the Parepro attribute sets when applied alone or in combination

Attribute set	Sensitivity	Specificity	Q2	MCC
RD	0.78	0.68	0.75	0.46
MI	0.79	0.66	0.74	0.46
IE	0.75	0.56	0.67	0.32
RD+MI	0.81	0.67	0.75	0.49
RD+IE	0.80	0.68	0.75	0.47
MI+IE	0.80	0.66	0.75	0.47
Parepro	0.82	0.67	0.76	0.50

Q2: the overall accuracy

MCC: Matthews correlation coefficient

### Effect of the number of homologous sequences on Parepro performance

**Figure 2:**
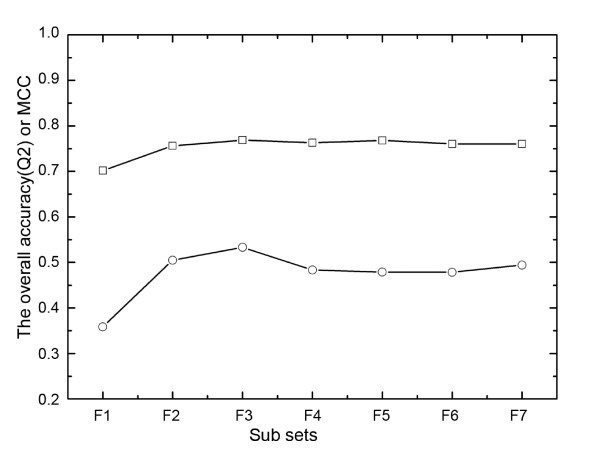
**The overall accuracy (Q2) and Matthews' correlation coefficient (MCC) of Parepro when testing the subsets from F1 to F7**. The *x*-axis denotes the different test subsets from F1 to F7, and the *y*-axis denotes the overall accuracy (Q2) or Matthews correlation coefficient (MCC).

To examine how the number of homologous sequences influenced the performance of Parepro, the HumVar dataset was split into seven sub datasets (i.e., F1, F2, F3, F4, F5, F6, F7) according to the number of homologous sequences, as summarized in Table 2. Then 20-fold cross-validation test was carried out on every sub datasets. Importantly, caution was taken to ensure that every test protein that contained the corresponding nsSNP was not included in the training set. As shown in Figure 2, the overall accuracy and MCC on sub dataset F1 were only about 70% and 36%, respectively. This result indicated that the prediction on the two classes (disease-related mutations and neutral polymorphisms) using sub dataset F1 was imbalance and only the major class obtained the better score. However, Parepro obtained the highest accuracy on sub dataset F3, which the overall accuracy (Q2) was 77% and the MCC was 54%. Therefore, these results indicated that the efficacy of Parepro for predicting amino acid variants depends on the number of homologous sequences.

**Table 2:** Range of the number of homologous sequences

Subset name	The range of homologous sequences number*	The proteins number within the range (%)	The mutations number within the range (%)
F1	[0,0]	12.29	8.28
F2	[1,3]	18.93	17.31
F3	[4,6]	11.84	9.27
F4	[7,9]	7.20	6.78
F5	[10,14]	9.70	10.65
F6	[15,25]	9.06	11.84
F7	[26,1000]	30.97	35.86

*The number of homologous sequences of target protein between a and b, as denoted by [a, b].

### Reliability index of Parepro for nsSNP prediction

**Figure 3:**
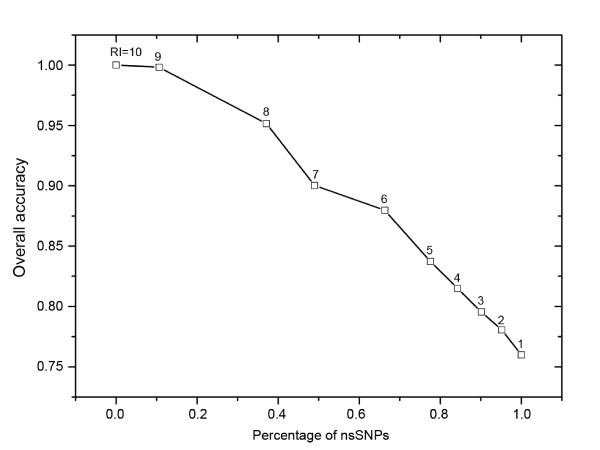
**Average prediction accuracy calculated cumulatively with RI above a given value**. For example, about 66% of all nsSNPs have RI ≥ 6, and of these nsNSPs about 88% are corrctly predicted. The result is based on the NumVar dataset.

When machine learning approaches are selected to predict the effects of nsSNPs on protein function, it is important to know the reliability of the predicted result [10,41]. In this study, a Reliability Index (RI) was also assigned to a predicted nsSNP based on the output of support vector machines that LIBSVM was used in this work. Consider that an output of LIBSVM with parameter of "-b 1" for a nsSNP is *O*; the RI value is thus computed as: *RI *= *INTEGER*(20 × *abs*(*O *- 0.5))+1. The RI assignment yields information about the certainty of the classification decision and thus can be used as an indicator of prediction certainty for a particular variant. Figure 3 shows the expected prediction accuracy and the proportion of the sequences with a given RI value. For example, about 66% of all nsSNPs had an RI ≥ 6, and of these nsSNPs about 88% were correctly predicted. These results are based on the HumVar dataset.

### Comparison of Parepro with other methods

We compared Parepro with other predictors, HybridMeth [10], PolyPhen [3] and SIFT [8,16,23]. HybridMeth uses the profile and sequence information surrounding a mutation. PolyPhen [3] is based on a decision tree and takes into account several pieces of information derived by structural parameters, functional annotations, and evolutionary information. SIFT [8,16,23] mainly uses information from homologous sequences.

As shown in Table 3, Parepro obtained the highest scores with respect to sensitivity, specificity, overall accuracy (Q2) and Matthews correlation coefficient (MCC) (the definition of these parameters could be find in method section) among the four methods. Because there was an obvious disparity in the number of disease-related mutations and the neutral polymorphisms in the dataset, MCC combined both the sensitivity and the specificity of the predictor and should be selected as the main score among the six scores in the evaluation [20,21,41,42]. The MCC for Parepro was higher by 6%, 17% and 4% compared with the MCC obtained with PolyPhen, SIFT and HydridMeth, respectively. Furthermore, Parepro could predict all mutations in the HumVar dataset. By contrast, PolyPhen and SIFT could only predict approximately 93% of the amino acid mutations, because these programs require more specific functional or evolutionary information. These results indicate that Parepro is a powerful tool for predicting the effect of mutations.

**Table 3:** Comparison of performance between Parepro and other methods using the HumVar dataset

Prediction Method	Sensitivity	Specificity	Q2	MCC	PM (%)
PolyPhen	0.62	0.80	0.72	0.44	93
SIFT	0.76	0.56	0.67	0.33	94
HydridMeth	0.80	0.65	0.74	0.46	100
Parepro	0.82	0.67	0.76	0.50	100

The prediction results of PolyPhen, SIFT and HydridMeth were obtained from Capriotti et al. [10].

Q2: the overall accuracy

MCC: Matthews correlation coefficient

PM is the percentage of predicted mutations.

### Predicted efficacy of Parepro on the NewHumVar dataset

To test the robustness of Parepro and compare it with other methods available on the web, the dataset NewHumVar was selected, which includes only new variants submitted to the Swiss-Prot database. Variants that were the same as in the HumVar dataset were removed. As shown in Table 4, all amino acid mutations in the NewHumVar dataset were predicted by Parepro. The MCC for Parepro was significantly higher than the MCCs calculated by HybridMeth, PolyPhen, and SIFT. These results indicate that Parepro outperformed these other prediction methods.

**Table 4:** Comparison of performance parameters of Parepro with other methods using the NewHumVar dataset

Prediction Method	Sensitivity	Specificity	Q2	MCC	PM (%)
PolyPhen	0.30	0.92	0.72	0.28	79
SIFT	0.32	0.87	0.69	0.22	88
HydridMeth	0.34	0.94	0.73	0.36	100
Parepro	0.40	0.94	0.78	0.42	100

The prediction results of PolyPhen, SIFT and HydridMeth were obtained from Capriotti et al. [10].

Q2: the overall accuracy

MCC: Matthews correlation coefficient

PM is the percentage of predicted mutations.

## Discussion

Predicting phenotypes resulting from nsSNPs is an important aspect of post-genome biology. The present study helps advance the analysis of genetic variation and may therefore lead to a better understanding of the resulting phenotypic variations among individuals with an aim toward drug design and development [2,7,20,25]. Two tests using different datasets indicated that Parepro outperformed several widely used methods.

Unlike the other methods that use the machine learning method [10,12,20-22,43,44], Parepro was constructed from three attribute sets RD, MI, and IE, all of which incorporate evolutionary information. In general, if the RD between the newly introduced amino acid and the residue in the mutation position has a high value, the substitution would be considered to have a high probability of being deleterious [16,18,25]. At the same time, attribute sets MI and IE were used to characterize the condition at the mutation position and around the mutation position, respectively. For example, when residues surrounding a mutation were found to be conserved, the region was related to either function or structure [10,27], and thus the mutation would be deleterious. This information reinforced the characterization provided by RD. Moreover, the results indicated that these three attribute sets complemented one another to yield a higher overall accuracy (Q2) and Matthews correlation coefficient(MCC).

The attribute vector of Parepro did not contain structural features. Thus, it is possible that some of the information directly derived from the protein structure [19] was ignored by Parepro. However, the lack of structural information was likely overcome by the inclusion of 50 discrete amino acid properties in the RD attribute set, thereby enhancing the efficacy of the sequence-based Parepro program.

## Conclusion

We present an SVM-based prediction method, Parepro, which predicts the effect of nsSNPs on protein function. Comprehensive comparisons of the prediction performance on two datasets showed that Parepro, which utilizes information from the amino acids surrounding the mutation position and from the residue difference between the newly introduced amino acid and the average residue in the mutation position, outperformed several other widely used prediction methods. Moreover, Parepro was able to predict all mutations within two distinct test sets. Therefore, we anticipate that Parepro will be a useful tool for large-scale analysis of nsSNPs in genomic databases.

## Methods

The prediction procedure of Parepro (Figure 1) begins by calculating the position-specific amino acid probabilities (PSAP) of a target protein that contains a corresponding nsSNP. Next, three attribute sets were constructed using PSAP and the properties of amino acids from AAindex [39,40]; these three sets were then used to describe residue differences (RD) and mutation position information (MI) and to yield information on the environment around the mutation positions (IE). Finally, a complex vector that consisted of 94 attributes was used to predict the effects of the nsSNPs. The attribute sets RD, MI and IE comprised 50, 23, 21 attributes, respectively.

### The mutation datasets

We used two datasets, HumVar and NewHumVar, taken from the PhD-SNP server [10]. The dataset HumVar consisted of 21,185 different SNPs (12,944 were disease-related, and 8,241 were neutral polymorphisms) obtained from 3,587 protein sequences in the Swiss-Prot database (Release 48). The NewHumVar dataset was comprised of SNPs obtained from the Swiss-Prot database (Release 50) after eliminating any variants also present in the HumVar dataset. Therefore, the dataset NewHumVar consisted of 935 single amino acid mutations (149 were disease-related variants, and 786 were neutral mutations) from 469 different proteins.

### Computing position-specific amino acid probabilities (PSAPs)

A Dirichlet mixture method [45-47] was adopted to estimate the PSAPs, which was then used to construct the vector of Parepro and was calculated as follows:

(1) PSI-BLAST [48] with parameter -e 0.001 was run for three iterations to collect sequences similar to the target protein that contained the corresponding nsSNP from the Swiss-Prot database (Release 50.0) [49]. The identified sequences were aligned by ClustalX [50,51] with default parameters. The position-based sequence weight method [52] was used to derive the weight *w*_*i *_of the *i*th sequence in the alignment. If no homologous sequence was selected, the weight *w*_*i *_of the target sequence was designated as 1.0.

(2) An alignment column was summarized by its weighted composition into a vector ***c***. The element of vector ***c ***was calculated as follows:

cm=∑i=1Nwi×δim(m=1,2⋯21)
 MathType@MTEF@5@5@+=feaafiart1ev1aaatCvAUfKttLearuWrP9MDH5MBPbIqV92AaeXatLxBI9gBaebbnrfifHhDYfgasaacPC6xNi=xI8qiVKYPFjYdHaVhbbf9v8qqaqFr0xc9vqFj0dXdbba91qpepeI8k8fiI+fsY=rqGqVepae9pg0db9vqaiVgFr0xfr=xfr=xc9adbaqaaeGacaGaaiaabeqaaeqabiWaaaGcbaGaem4yam2aaSbaaSqaaiabd2gaTbqabaGccqGH9aqpdaaeWbqaaiabdEha3naaBaaaleaacqWGPbqAaeqaaOGaey41aqlcciGae8hTdq2aaSbaaSqaaiabdMgaPjabd2gaTbqabaaabaGaemyAaKMaeyypa0JaeGymaedabaGaemOta4eaniabggHiLdGccqGGOaakcqWGTbqBcqGH9aqpcqaIXaqmcqGGSaalcqaIYaGmcqWIVlctcqaIYaGmcqaIXaqmcqGGPaqkaaa@4B28@

where *N *is the total number of aligned sequences, *w*_*i *_is the weight of the *i *th sequence, the value of *m *from 1 to 20 represents any one of 20 amino acids, and a value of 21 represents a gap. If the symbol type of the *i *th sequence at the column is an amino acid *a*_*m*_(*m *= 1, 2⋯20) or gap (*m *= 21), the value of *δ*_*im *_is 1.0; otherwise it is 0.

(3) A new vector ***u***, which incorporated the gap information into the 20 amino acids, was constructed as follows:

*u*_*m *_= *c*_*m *_+ *c*_21 _× *h*_*m *_(*m *= 1, 2⋯20)

where the vector *h *is the frequency of occurrence of any one of the 20 amino acids [53].

(4) The Dirichlet mixture method [45-47] was adopted to estimate the PSAPs. The posterior probability of amino acid *m *at a position, p^m
 MathType@MTEF@5@5@+=feaafiart1ev1aaatCvAUfKttLearuWrP9MDH5MBPbIqV92AaeXatLxBI9gBaebbnrfifHhDYfgasaacPC6xNi=xH8viVGI8Gi=hEeeu0xXdbba9frFj0xb9qqpG0dXdb9aspeI8k8fiI+fsY=rqGqVepae9pg0db9vqaiVgFr0xfr=xfr=xc9adbaqaaeGacaGaaiaabeqaaeqabiWaaaGcbaGafmiCaaNbaKaadaWgaaWcbaGaemyBa0gabeaaaaa@2EDB@, was calculated from a 20-component Dirichlet mixture[47]:

p^m=Xm∑k=120Xk(m=1,2⋯20)
 MathType@MTEF@5@5@+=feaafiart1ev1aaatCvAUfKttLearuWrP9MDH5MBPbIqV92AaeXatLxBI9gBaebbnrfifHhDYfgasaacPC6xNi=xI8qiVKYPFjYdHaVhbbf9v8qqaqFr0xc9vqFj0dXdbba91qpepeI8k8fiI+fsY=rqGqVepae9pg0db9vqaiVgFr0xfr=xfr=xc9adbaqaaeGacaGaaiaabeqaaeqabiWaaaGcbaGafmiCaaNbaKaadaWgaaWcbaGaemyBa0gabeaakiabg2da9KqbaoaalaaabaGaemiwaG1aaSbaaeaacqWGTbqBaeqaaaqaamaaqahabaGaemiwaG1aaSbaaeaacqWGRbWAaeqaaaqaaiabdUgaRjabg2da9iabigdaXaqaaiabikdaYiabicdaWaGaeyyeIuoaaaGcdaqadaqaaiabd2gaTjabg2da9iabigdaXiabcYcaSiabikdaYiabl+UimjabikdaYiabicdaWaGaayjkaiaawMcaaaaa@4838@

Xm=∑j=1lqjB(α→j+n→)B(α→j)×αj,m+nm|a→j|+|n→|
 MathType@MTEF@5@5@+=feaafiart1ev1aaatCvAUfKttLearuWrP9MDH5MBPbIqV92AaeXatLxBI9gBaebbnrfifHhDYfgasaacPC6xNi=xI8qiVKYPFjYdHaVhbbf9v8qqaqFr0xc9vqFj0dXdbba91qpepeI8k8fiI+fsY=rqGqVepae9pg0db9vqaiVgFr0xfr=xfr=xc9adbaqaaeGacaGaaiaabeqaaeqabiWaaaGcbaGaemiwaG1aaSbaaSqaaiabd2gaTbqabaGccqGH9aqpdaaeWbqaaiabdghaXnaaBaaaleaacqWGQbGAaeqaaKqbaoaalaaabaGaemOqaiKaeiikaGccciGaf8xSdeMbaSaadaWgaaqaaiabdQgaQbqabaGaey4kaSIafmOBa4MbaSaacqGGPaqkaeaacqWGcbGqcqGGOaakcuWFXoqygaWcamaaBaaabaGaemOAaOgabeaacqGGPaqkaaGccqGHxdaTjuaGdaWcaaqaaiab=f7aHnaaBaaabaGaemOAaOMaeiilaWIaemyBa0gabeaacqGHRaWkcqWGUbGBdaWgaaqaaiabd2gaTbqabaaabaWaaqWaaeaacuWGHbqygaWcamaaBaaabaGaemOAaOgabeaaaiaawEa7caGLiWoacqGHRaWkdaabdaqaaiqbd6gaUzaalaaacaGLhWUaayjcSdaaaaWcbaGaemOAaOMaeyypa0JaeGymaedabaGaemiBaWganiabggHiLdaaaa@6015@

where *q*_*j *_is the mixture coefficient of each component, *B *is the Beta function, α→j
 MathType@MTEF@5@5@+=feaafiart1ev1aaatCvAUfKttLearuWrP9MDH5MBPbIqV92AaeXatLxBI9gBaebbnrfifHhDYfgasaacPC6xNi=xH8viVGI8Gi=hEeeu0xXdbba9frFj0xb9qqpG0dXdb9aspeI8k8fiI+fsY=rqGqVepae9pg0db9vqaiVgFr0xfr=xfr=xc9adbaqaaeGacaGaaiaabeqaaeqabiWaaaGcbaacciGaf8xSdeMbaSaadaWgaaWcbaGaemOAaOgabeaaaaa@2F14@ = (*α*_*j*1_...*α*_*j*20_) is the parameter for each component *j *of the Dirichlet mixture, and *l *is the number of components. The vector ***n ***was calculated by the equation, *n*_*m *_= *u*_*m*_× *N*(*m *= 1, 2⋯20), where *N *is the total number of homologous sequences and *u*_*m*_is calculated from equation (2).

### Inputs and Encoding Schemes of Parepro

The Parepro vector was comprised of three attribute sets, which were used to describe the RD, the MI, and the IE.

The first attribute set, RD, was designed to depict the property differences between the newly introduced amino acid and the average residue in the mutation position, which was composed of 50 elements and was constructed as follows:

(1) The 544 amino acid properties were downloaded from AAindex [39,40], as shown in Additional file 1. Then the value of each property *t*_*km*_(*k *= 1,⋯,544, *m *= 1, 2⋯20) was standardized as follows:

tkm'=tkm−μksk
 MathType@MTEF@5@5@+=feaafiart1ev1aaatCvAUfKttLearuWrP9MDH5MBPbIqV92AaeXatLxBI9gBaebbnrfifHhDYfgasaacPC6xNi=xI8qiVKYPFjYdHaVhbbf9v8qqaqFr0xc9vqFj0dXdbba91qpepeI8k8fiI+fsY=rqGqVepae9pg0db9vqaiVgFr0xfr=xfr=xc9adbaqaaeGacaGaaiaabeqaaeqabiWaaaGcbaGaemiDaq3aa0baaSqaaiabdUgaRjabd2gaTbqaaiabcEcaNaaakiabg2da9KqbaoaalaaabaGaemiDaq3aaSbaaeaacqWGRbWAcqWGTbqBaeqaaiabgkHiTGGaciab=X7aTnaaBaaabaGaem4AaSgabeaaaeaacqWGZbWCdaWgaaqaaiabdUgaRbqabaaaaaaa@3E72@

where *μ*_*k *_and sk2
 MathType@MTEF@5@5@+=feaafiart1ev1aaatCvAUfKttLearuWrP9MDH5MBPbIqV92AaeXatLxBI9gBaebbnrfifHhDYfgasaacPC6xNi=xH8viVGI8Gi=hEeeu0xXdbba9frFj0xb9qqpG0dXdb9aspeI8k8fiI+fsY=rqGqVepae9pg0db9vqaiVgFr0xfr=xfr=xc9adbaqaaeGacaGaaiaabeqaaeqabiWaaaGcbaGaem4Cam3aa0baaSqaaiabdUgaRbqaaiabikdaYaaaaaa@2FC0@ are the mean and variance of the property *k*, respectively, and were calculated as follows: μk=120∑m=120tkm
 MathType@MTEF@5@5@+=feaafiart1ev1aaatCvAUfKttLearuWrP9MDH5MBPbIqV92AaeXatLxBI9gBaebbnrfifHhDYfgasaacPC6xNi=xH8viVGI8Gi=hEeeu0xXdbba9frFj0xb9qqpG0dXdb9aspeI8k8fiI+fsY=rqGqVepae9pg0db9vqaiVgFr0xfr=xfr=xc9adbaqaaeGacaGaaiaabeqaaeqabiWaaaGcbaacciGae8hVd02aaSbaaSqaaiabdUgaRbqabaGccqGH9aqpjuaGdaWcaaqaaiabigdaXaqaaiabikdaYiabicdaWaaakmaaqahabaGaemiDaq3aaSbaaSqaaiabdUgaRjabd2gaTbqabaaabaGaemyBa0Maeyypa0JaeGymaedabaGaeGOmaiJaeGimaadaniabggHiLdaaaa@3F72@ and sk2=119∑m=120(tkm−μk)2
 MathType@MTEF@5@5@+=feaafiart1ev1aaatCvAUfKttLearuWrP9MDH5MBPbIqV92AaeXatLxBI9gBaebbnrfifHhDYfgasaacPC6xNi=xH8viVGI8Gi=hEeeu0xXdbba9frFj0xb9qqpG0dXdb9aspeI8k8fiI+fsY=rqGqVepae9pg0db9vqaiVgFr0xfr=xfr=xc9adbaqaaeGacaGaaiaabeqaaeqabiWaaaGcbaGaem4Cam3aa0baaSqaaiabdUgaRbqaaiabikdaYaaakiabg2da9KqbaoaalaaabaGaeGymaedabaGaeGymaeJaeGyoaKdaaOWaaabCaeaacqGGOaakcqWG0baDdaWgaaWcbaGaem4AaSMaemyBa0gabeaakiabgkHiTGGaciab=X7aTnaaBaaaleaacqWGRbWAaeqaaOGaeiykaKYaaWbaaSqabeaacqaIYaGmaaaabaGaemyBa0Maeyypa0JaeGymaedabaGaeGOmaiJaeGimaadaniabggHiLdaaaa@4741@.

(2) The position-dependent properties *d*_*k *_were given by

dkm=pm×tkm'
 MathType@MTEF@5@5@+=feaafiart1ev1aaatCvAUfKttLearuWrP9MDH5MBPbIqV92AaeXatLxBI9gBaebbnrfifHhDYfgasaacPC6xNi=xI8qiVKYPFjYdHaVhbbf9v8qqaqFr0xc9vqFj0dXdbba91qpepeI8k8fiI+fsY=rqGqVepae9pg0db9vqaiVgFr0xfr=xfr=xc9adbaqaaeGacaGaaiaabeqaaeqabiWaaaGcbaGaemizaq2aaSbaaSqaaiabdUgaRjabd2gaTbqabaGccqGH9aqpcqWGWbaCdaWgaaWcbaGaemyBa0gabeaakiabgEna0kabdsha0naaDaaaleaacqWGRbWAcqWGTbqBaeaacqGGNaWjaaaaaa@3BBF@

where *p*_*m *_is the PSAP at a mutated position calculated from equation (3).

(3) With respect to property *k*, the distance *r*_*k *_between the weighted property *d*_*kn *_of a newly introduced amino acid *n *and the mean of *d*_*k *_was

rk=dkn−μk'sk'
 MathType@MTEF@5@5@+=feaafiart1ev1aaatCvAUfKttLearuWrP9MDH5MBPbIqV92AaeXatLxBI9gBaebbnrfifHhDYfgasaacPC6xNi=xI8qiVKYPFjYdHaVhbbf9v8qqaqFr0xc9vqFj0dXdbba91qpepeI8k8fiI+fsY=rqGqVepae9pg0db9vqaiVgFr0xfr=xfr=xc9adbaqaaeGacaGaaiaabeqaaeqabiWaaaGcbaGaemOCai3aaSbaaSqaaiabdUgaRbqabaGccqGH9aqpjuaGdaWcaaqaaiabdsgaKnaaBaaabaGaem4AaSMaemOBa4gabeaacqGHsisliiGacqWF8oqBdaqhaaqaaiabdUgaRbqaaiabcEcaNaaaaeaacqWGZbWCdaqhaaqaaiabdUgaRbqaaiabcEcaNaaaaaaaaa@3DC4@

where μk'
 MathType@MTEF@5@5@+=feaafiart1ev1aaatCvAUfKttLearuWrP9MDH5MBPbIqV92AaeXatLxBI9gBaebbnrfifHhDYfgasaacPC6xNi=xH8viVGI8Gi=hEeeu0xXdbba9frFj0xb9qqpG0dXdb9aspeI8k8fiI+fsY=rqGqVepae9pg0db9vqaiVgFr0xfr=xfr=xc9adbaqaaeGacaGaaiaabeqaaeqabiWaaaGcbaacciGae8hVd02aa0baaSqaaiabdUgaRbqaaiabcEcaNaaaaaa@2FF2@ and sk'2
 MathType@MTEF@5@5@+=feaafiart1ev1aaatCvAUfKttLearuWrP9MDH5MBPbIqV92AaeXatLxBI9gBaebbnrfifHhDYfgasaacPC6xNi=xH8viVGI8Gi=hEeeu0xXdbba9frFj0xb9qqpG0dXdb9aspeI8k8fiI+fsY=rqGqVepae9pg0db9vqaiVgFr0xfr=xfr=xc9adbaqaaeGacaGaaiaabeqaaeqabiWaaaGcbaGaem4Cam3aa0baaSqaaiabdUgaRbqaaiabcEcaNiabikdaYaaaaaa@3096@ are the mean and variance of *d*_*k*_, respectively, and were calculated as follows: μk'=120∑m=120dkm
 MathType@MTEF@5@5@+=feaafiart1ev1aaatCvAUfKttLearuWrP9MDH5MBPbIqV92AaeXatLxBI9gBaebbnrfifHhDYfgasaacPC6xNi=xH8viVGI8Gi=hEeeu0xXdbba9frFj0xb9qqpG0dXdb9aspeI8k8fiI+fsY=rqGqVepae9pg0db9vqaiVgFr0xfr=xfr=xc9adbaqaaeGacaGaaiaabeqaaeqabiWaaaGcbaacciGae8hVd02aa0baaSqaaiabdUgaRbqaaiabcEcaNaaakiabg2da9KqbaoaalaaabaGaeGymaedabaGaeGOmaiJaeGimaadaaOWaaabCaeaacqWGKbazdaWgaaWcbaGaem4AaSMaemyBa0gabeaaaeaacqWGTbqBcqGH9aqpcqaIXaqmaeaacqaIYaGmcqaIWaama0GaeyyeIuoaaaa@4029@, sk'2=119∑m=120(dkm−μk')2
 MathType@MTEF@5@5@+=feaafiart1ev1aaatCvAUfKttLearuWrP9MDH5MBPbIqV92AaeXatLxBI9gBaebbnrfifHhDYfgasaacPC6xNi=xH8viVGI8Gi=hEeeu0xXdbba9frFj0xb9qqpG0dXdb9aspeI8k8fiI+fsY=rqGqVepae9pg0db9vqaiVgFr0xfr=xfr=xc9adbaqaaeGacaGaaiaabeqaaeqabiWaaaGcbaGaem4Cam3aa0baaSqaaiabdUgaRbqaaiabcEcaNiabikdaYaaakiabg2da9KqbaoaalaaabaGaeGymaedabaGaeGymaeJaeGyoaKdaaOWaaabCaeaacqGGOaakcqWGKbazdaWgaaWcbaGaem4AaSMaemyBa0gabeaakiabgkHiTGGaciab=X7aTnaaDaaaleaacqWGRbWAaeaacqGGNaWjaaGccqGGPaqkdaahaaWcbeqaaiabikdaYaaaaeaacqWGTbqBcqGH9aqpcqaIXaqmaeaacqaIYaGmcqaIWaama0GaeyyeIuoaaaa@48CE@.

(4) A new vector *r *was then constructed using the 544 elements from Additional file 1. The software weka3.4 [54] was used to simplify the vector *r*, in which the evaluator CfsSubsetEvalwas selected. The redundant and low-contribution elements in vector *r *were removed. After these modifications, 50 elements remained and were included in the RD attribute set.

The second attribute set, MI, was used to define the status of a mutation position and consisted of 23 values. The first 20 elements were the PSAP values of the 20 amino acids in the mutation position calculated from equation (3). The 21^st ^and 22^nd ^elements were the PSAP values of the wild-type residue and the newly introduced residue, respectively. The 23^rd ^value was the entropy (*E*) [55,56] of amino acids in the mutation position and was calculated as follows:

E=−1ln⁡20∑m=120pmln⁡pm
 MathType@MTEF@5@5@+=feaafiart1ev1aaatCvAUfKttLearuWrP9MDH5MBPbIqV92AaeXatLxBI9gBaebbnrfifHhDYfgasaacPC6xNi=xI8qiVKYPFjYdHaVhbbf9v8qqaqFr0xc9vqFj0dXdbba91qpepeI8k8fiI+fsY=rqGqVepae9pg0db9vqaiVgFr0xfr=xfr=xc9adbaqaaeGacaGaaiaabeqaaeqabiWaaaGcbaGaemyrauKaeyypa0JaeyOeI0scfa4aaSaaaeaacqaIXaqmaeaacyGGSbaBcqGGUbGBcqaIYaGmcqaIWaamaaGcdaaeWbqaaiabdchaWnaaBaaaleaacqWGTbqBaeqaaOGagiiBaWMaeiOBa4MaemiCaa3aaSbaaSqaaiabd2gaTbqabaaabaGaemyBa0Maeyypa0JaeGymaedabaGaeGOmaiJaeGimaadaniabggHiLdaaaa@4595@

where 20 is the number of amino acids, and *p*_*m *_is the PSAP value at the mutation position calculated from equation (3).

The third attribute set, IE, encoded the information surrounding the mutation position and consisted of 21 elements. The first 20 elements represented the PSAP values of the 20 amino acids and were calculated from equation (3), and the last element represented entropy and was calculated from equation (8). Residues in the immediate vicinity of the mutation carried more significance with respect to the mutation. Therefore, a significance coefficient was assigned to each residue in proximity to the mutation. The element of IE was then calculated as follows:

IEa=1f+1×∑m=−fff+1−abs(m)f+1y(i+m)a(a=1,2⋯21)
 MathType@MTEF@5@5@+=feaafiart1ev1aaatCvAUfKttLearuWrP9MDH5MBPbIqV92AaeXatLxBI9gBaebbnrfifHhDYfgasaacPC6xNi=xI8qiVKYPFjYdHaVhbbf9v8qqaqFr0xc9vqFj0dXdbba91qpepeI8k8fiI+fsY=rqGqVepae9pg0db9vqaiVgFr0xfr=xfr=xc9adbaqaaeGacaGaaiaabeqaaeqabiWaaaGcbaGaemysaKKaemyrau0aaSbaaSqaaiabdggaHbqabaGccqGH9aqpjuaGdaWcaaqaaiabigdaXaqaaiabdAgaMjabgUcaRiabigdaXaaakiabgEna0oaaqahabaqcfa4aaSaaaeaacqWGMbGzcqGHRaWkcqaIXaqmcqGHsislcqWGHbqycqWGIbGycqWGZbWCcqGGOaakcqWGTbqBcqGGPaqkaeaacqWGMbGzcqGHRaWkcqaIXaqmaaGccqWG5bqEdaWgaaWcbaGaeiikaGIaemyAaKMaey4kaSIaemyBa0MaeiykaKIaemyyaegabeaakiabcIcaOiabdggaHjabg2da9iabigdaXiabcYcaSiabikdaYiabl+UimjabikdaYiabigdaXiabcMcaPaWcbaGaemyBa0Maeyypa0JaeyOeI0IaemOzaygabaGaemOzayganiabggHiLdaaaa@61D0@

where *i *is the mutation position, *f *is the number of residues located to the left or right of the mutation position, and *a *represents one element of IE from 1 to 21. If the value of *a *is between 1 and 20, *y*_(*i*+*m*)*a *_is *p*_*a *_in the position of *i *+ *m *calculated from equation (3). However, if the value of *a *is 21, *y*_(*i*+*m*)*a *_is the entropy *E*_*i*+*m *_calculated from equation (8). Furthermore, if the mutation is located at the N-terminal position (*i *+ *m *> *l*) or at the C-terminal position, then *y*_(*i*+*m*)*a *_is *y*_*la *_or *y*_*la*_, respectively, where *l *is the number of residues in the protein.

### Support vector machine

The SVM is a classifier seeking an optimal hyperplane to separate two classes of samples. SVM uses kernel functions to map original data to a feature space of higher dimensions and locates an optimal separating hyperplane. For SVM implementation, we used LIBSVM [57] with a Radial Basis Function (RBF kernel function) *K*(*x*_*i*_, *x*_*j*_) = exp(-*G*||*x*_*i *_- *x*_*j*_||^2^). The parameter was selected with the LIBSVM parameter selection tool.

### Scoring the performance

The proteins in the dataset were randomly divided into 20 subsets. For each individual test, the mutations in one of the 20 sub-datasets were used as the test set and the others in the 19 subsets were combined to form a training set. The procedure was repeated 20 times so that each sample was used exactly once for testing and training. We defined disease-associated nsSNPs as positive and neutral nsSNPs as negative. In this work, we adopted sensitivity, specificity, overall accuracy(Q2) and Matthews correlation coefficient(MCC) to score the performance of the corresponding method:

Sencitivity=TPTP+FN,Specificity=TNTN+FP,Q2=TP+TNTP+FP+TN+FNMCC=TP×TN−FP×FN(TN+FN)×(TN+FP)×(TP+FN)×(TP+FP)
 MathType@MTEF@5@5@+=feaafiart1ev1aaatCvAUfKttLearuWrP9MDH5MBPbIqV92AaeXatLxBI9gBaebbnrfifHhDYfgasaacPC6xNi=xI8qiVKYPFjYdHaVhbbf9v8qqaqFr0xc9vqFj0dXdbba91qpepeI8k8fiI+fsY=rqGqVepae9pg0db9vqaiVgFr0xfr=xfr=xc9adbaqaaeGacaGaaiaabeqaaeqabiWaaaGcbaqbaeaabiqaaaqaauaabeqabmaaaeaacqWGtbWucqWGLbqzcqWGUbGBcqWGJbWycqWGPbqAcqWG0baDcqWGPbqAcqWG2bGDcqWGPbqAcqWG0baDcqWG5bqEcqGH9aqpjuaGdaWcaaqaaiabdsfaujabdcfaqbqaaiabdsfaujabdcfaqjabgUcaRiabdAeagjabd6eaobaakiabcYcaSaqaaiabdofatjabdchaWjabdwgaLjabdogaJjabdMgaPjabdAgaMjabdMgaPjabdogaJjabdMgaPjabdsha0jabdMha5jabg2da9KqbaoaalaaabaGaemivaqLaemOta4eabaGaemivaqLaemOta4Kaey4kaSIaemOrayKaemiuaafaaOGaeiilaWcabaGaemyuaeLaeGOmaiJaeyypa0tcfa4aaSaaaeaacqWGubavcqWGqbaucqGHRaWkcqWGubavcqWGobGtaeaacqWGubavcqWGqbaucqGHRaWkcqWGgbGrcqWGqbaucqGHRaWkcqWGubavcqWGobGtcqGHRaWkcqWGgbGrcqWGobGtaaaaaaGcbaGaemyta0Kaem4qamKaem4qamKaeyypa0tcfa4aaSaaaeaacqWGubavcqWGqbaucqGHxdaTcqWGubavcqWGobGtcqGHsislcqWGgbGrcqWGqbaucqGHxdaTcqWGgbGrcqWGobGtaeaadaGcaaqaaiabcIcaOiabdsfaujabd6eaojabgUcaRiabdAeagjabd6eaojabcMcaPiabgEna0kabcIcaOiabdsfaujabd6eaojabgUcaRiabdAeagjabdcfaqjabcMcaPiabgEna0kabcIcaOiabdsfaujabdcfaqjabgUcaRiabdAeagjabd6eaojabcMcaPiabgEna0kabcIcaOiabdsfaujabdcfaqjabgUcaRiabdAeagjabdcfaqjabcMcaPaqabaaaaaaaaaa@AA30@

where TP is the number of true positives, TN is the number of true negatives, FP is the number of false positives, and FN is the number of false negatives. Because there was an obvious disparity in the number of positive samples and negative samples in the dataset, MCC combined both the sensitivity and the specificity of the predictor and should be selected as the main score among the six scores in the evaluation [20,21,41,42].

## Availability and requirements

Project name: Parepro

Project home page: 

Operating systems: Windows

Programming language: Perl

License: GNU General Public License. This license allows the source code to be redistributed and/or modified under the terms of the GNU General Public License as published by the Free Software Foundation. The source code for the application is available at no charge.

Any restrictions to use by non-academics: None

## Authors' contributions

Jian Tian wrote the code of Parepro. Ningfeng Wu, Juhua Zhang and Yunliu Fan supervised the work. Jian Tian, Ningfeng Wu, Xuexia Guo and Jun Guo were involved in the preparation of the manuscript. Jian Tian, Ningfeng Wu, Xuexia Guo, Jun Guo, Juhua Zhang and Yunliu Fan read and approved the manuscript.

## Supplementary Material

Additional file 1 The amino acid properties used in Parepro. The file can be viewed by the software Excel. Click here for file
